# BRD2 impedes iPSC reprogramming by regulating lipogenesis and matrisome

**DOI:** 10.21203/rs.3.rs-7744310/v1

**Published:** 2025-12-05

**Authors:** Ricardo R Cevallos, Ruowen Zhang, Shu G. Chen, Kejin Hu

**Affiliations:** 1, Department of Microbiology, Immunology and Genetics, University of North Texas Health Science Center, Fort Worth, Texas, 76107; 2, Department of Pathology, University of Alabama at Birmingham, AL; 3, Department of Biochemistry and Molecular Genetics, University of Alabama at Birmingham, AL

## Abstract

Induction of human pluripotent stem cells (HiPSCs) from somatic cells encounters significant barriers, which remain poorly understood. Lipids are fundamental cellular molecules with many essential roles. However, lipid roles in reprogramming are unknown. Here, we prove that BRD2 is a barrier to HiPSC reprogramming by suppressing lipogenesis and maintaining the somatic transcriptional program, particularly the somatic matrisome program. Strikingly, the acetylation epigenetic reader BRD2 unorthodoxically suppressed gene expression of lipogenesis in the reprogramming cells. The two rate-limiting enzymes of lipogenesis, SCD and HMGCR, enhanced iPSC reprogramming while lipid supplements enhanced it. Interestingly, the BRD2 ET tail suppressed reprogramming and the lipogenesis transcriptional program but positively regulated matrisome program. In line with its transcriptional suppression of lipogenesis, BRD2 binds to genes of lipogenesis and negatively regulates their H3K27Ac status. These discoveries advance our knowledge of HiPSC reprogramming by revealing the opposite BRD2 regulatory roles on matrisome and lipogenesis in promoting reprogramming.

## Introduction

Human somatic cells can be converted into embryonic stem cell- (ESC-)like cells by the Yamanaka factors, OCT4, SOX2, KLF4 and MYC (collectively OSKM)^7–9^. Such factor-induced pluripotent stem cells (iPSCs) share molecular and functional characteristics with ESCs and are great resources for basic research, regenerative medicine, drugs screening, disease modeling, and others^10,11^. HiPSC reprogramming, however, is very inefficient, slow, inconsistent, and stochastic, with significant barriers^1,12^. Past investigation has revealed many reprogramming barriers such as DNA methylation, somatic transcription factors, and others. Our molecular understandings about iPSC reprogramming, particularly its molecular barriers, remains limited. It is well-known that culture of human PSCs (hPSCs) uniquely requires supports of feeder-cells^13^ or protein matrix precoated on the culture vessels^14^, indicating that hPSCs have a unique matrix biology^15^, and thus matrisome reprogramming may be critical for HiPSC generation.

Bromodomain extra terminal (BET) proteins are characterized by two N-terminal tandem bromodomains (BD) and one extra terminal (ET) domain in their C-terminal tails. BET proteins bind to acetylated lysine in histones via their bromodomains and thus regulate transcription^16^. Although BET proteins are known as positive regulators of transcription, suppression of transcription has also been observed^17,18^. Mammalian BET proteins include BRD2, BRD3, BRD4 and BRDt, with the first three as ubiquitous proteins. BET proteins regulate a variety of cellular and physiological processes including transcription, cell cycles, tumorigenesis, inflammation, diabetics, stemness, and others^19,20^. They are implicated in cellular reprogramming^17,21–26^, but the exact underlying molecular mechanisms remain elusive. Furthermore, the roles of each member are not defined. Liu *et al*., reported that BRD4 enhanced mouse iPSC reprogramming^24^ but Zhang *et al*. showed that BRD4 inhibition facilitated it^25^. We previously reported that mild pharmaceutical BET inhibition enhanced HiPSC generation and dampened somatic transcription^17^. In line with our observation, Li *et al*. showed that chemical BET inhibition promoted mouse fibroblast reprogramming to functional neurons likely by suppressing somatic transcription^23^.

Lipids are one of the four fundamental cellular molecules among proteins, nucleic acids and carbohydrates. Humans may have more than 800 species of lipids^27^. They are basic cellular structural molecules essential for all biological membranes. Lipids are also cellular energy sources, nutrients, hormones, and signaling molecules as well as protein modification elements^2–4^. The complex lipogenesis involves multiple pathways and many enzymes. Of those, stearoyl-CoA desaturase (SCD) catalyzes generation of the first double bond as the rate-limiting step in the fundamental fatty acid (FA) biosynthesis^5^. At the same time, HMG-CoA reductase (HMGCR) regulates the rate-limiting step of cholesterol biosynthesis^6^. *De novo* lipogenesis is a characteristic feature of naïve mammalian PSCs, and of the traditional hPSCs cultured in the chemically defined E8 media^28,29^, implying a need for lipogenesis reprogramming as part of the pluripotency establishment. However, no study has ever been carried out about the role of lipogenesis in iPSC reprogramming.

Here, we report that BRD2 is a major HiPSC reprogramming barrier. We first showed that BRD2 may impair iPSC reprogramming by maintaining somatic transcription, particularly for genes of matrisome and its regulation. Surprisingly, we discovered that BRD2, known as a positive transcriptional regulator, unconventionally suppresses genes in lipogenesis and lipid metabolism likely by direct binding to those genes and regulating their epigenetic status. Critically, we showed that key lipogenesis enzymes promote iPSC reprogramming and that lipid supplements into the reprogramming media can overcome lipogenesis deficiency during reprogramming.

## Results

### Human BRD2 is an early barrier of iPSC reprogramming

**Extended Data Fig. 1 |:**
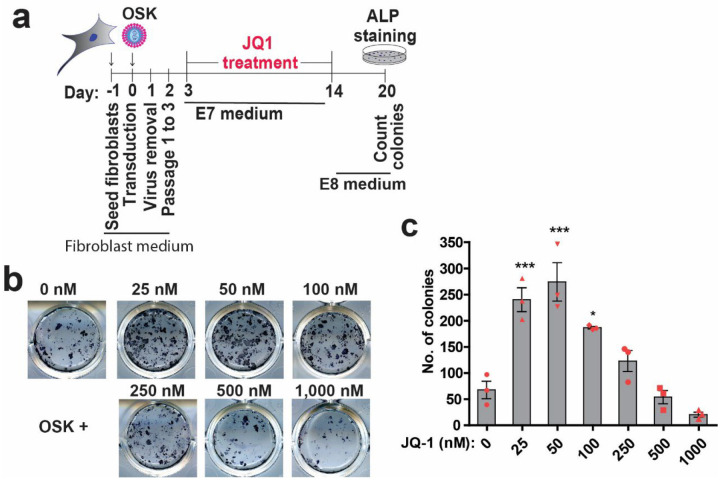
BET inhibitor JQ1 enhances reprogramming at lower concentrations (related to Fig. 1). **a**, Schematic for confirming roles of BET inhibition on human iPSC reprogramming. JQ1 of various concentration was included from day 3 to 14 of reprogramming and colonies positive for alkaline phosphatase (ALP) activity were counted at day 20 post transduction. **b**, Shown are representative images of ALP-stained wells on day 20 of reprogramming. **c**, Total colony counts per well of a 24-well plate on day 20. Mean and SD are shown (ANOVA, n = 3; *, p < 0.05; ***, p < 0.001).

**Fig. 1 |:**
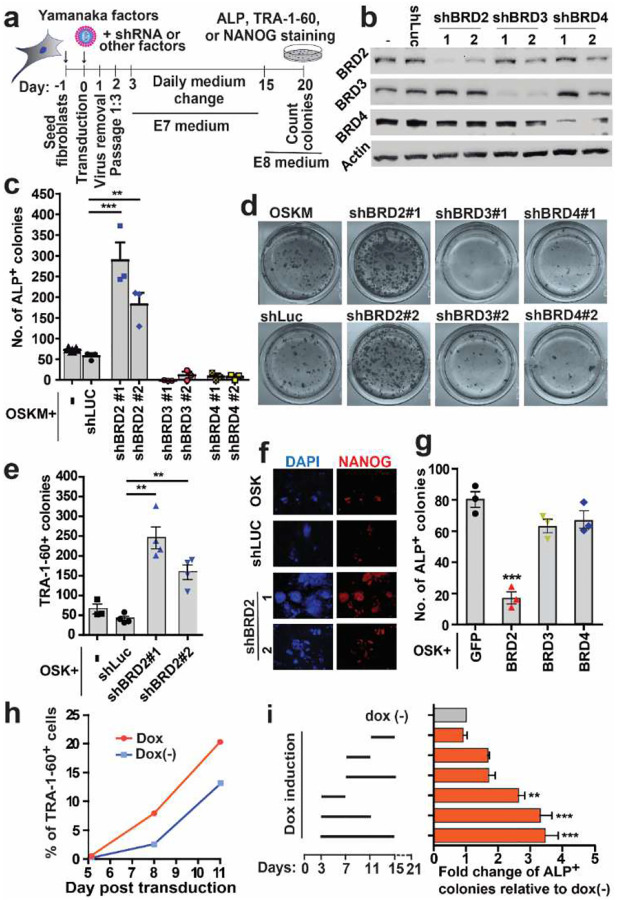
Human BRD2 is an early reprogramming barrier **a**, Schematic for evaluation of the reprogramming efficiency in the clinic-relevant chemically defined medium. **b,** Western blots on human fibroblasts with the lentiviral shRNAs against human *BRD2*, *BRD3* and *BRD4*. Two shRNA vectors (1 and 2) were used for each BET member. Beta actin was probed as a protein loading control. **c,** Average total ALP^+^ colony counts of three independent experiments at day 20 of OSKM reprogramming using the shRNAs shown in B. n = 3; **, p<0.001; ***, p < 0.0001. t-test. **d**, Representative images of alkaline phosphatase staining of experiments shown in C. **e**, Average colony counts of TRA-1–60 positive colonies in three independent OSK reprogramming experiments. **f**, Representative images of colonies expressing NANOG at day 20 of OSK reprogramming experiments. **g**, Average total ALP^+^ colony counts at day 20 of three independent OSK reprogramming experiments with the overexpression of BET proteins. **h**, Percentage of TRA-1–60 positive cells at days 5, 8 and 11 during reprogramming using a doxycycline (dox) inducible shBRD2 vector as measured by flow cytometry. **i**, Fold changes of ALP^+^ colonies under different times of BRD2 knockdown relative to no knockdown control using the dox inducible shBRD2 vector. Dox was added at different time windows during the first 15 days of OSK reprogramming. n = 3 (independent experiments); *, p < 0.05; ANOVA.

We previously reported that chemical inhibition of BET proteins using the widely used concentrations (e.g., >= 500 nM for JQ1) had little impact on or even impaired HiPSC reprogramming^21^, but their mild pharmacological inhibition improved it^17^. The present study confirmed our previous observations (Extended Data Fig. 1). Mammalian BET family has three ubiquitously expressed members BRD2, BRD3 and BRD4, all of which are embryonic lethal when knocked out individually (BRD3 embryonic lethality is based on our unpublished observation)^30,31^, indicating their essential but distinct roles. The current bromodomain-targeting BET chemical inhibitors do not distinguish individual members^32^. Given the above facts, we asked whether mild BET chemical inhibition targets a specific BET member to promote HiPSC reprogramming. We first established two efficient shRNAs against each BET member (Fig. 1b). Reprogramming experiments (Fig. 1a) showed that *BRD3* and *BRD4* shRNAs compromised HiPSC generation under the OSKM condition (Fig. 1c and 1d). In contrast, both shRNAs of human *BRD2* significantly enhanced HiPSC reprogramming (Fig. 1c and 1d). Similar results were observed under the Yamanaka 3- factor (OSK) condition (Fig. 1e). We verified our observations using other pluripotency markers (TRA-1–60 and NANOG, Fig. 1e, 1f and 1h). In agreement with *BRD2* shRNA knockdown, *BRD2* overexpression significantly decreased the reprogramming while overexpression of *BRD3* and *BRD4S* had little impact on reprogramming (Fig. 1g).

**Extended Data Fig. 2 |:**
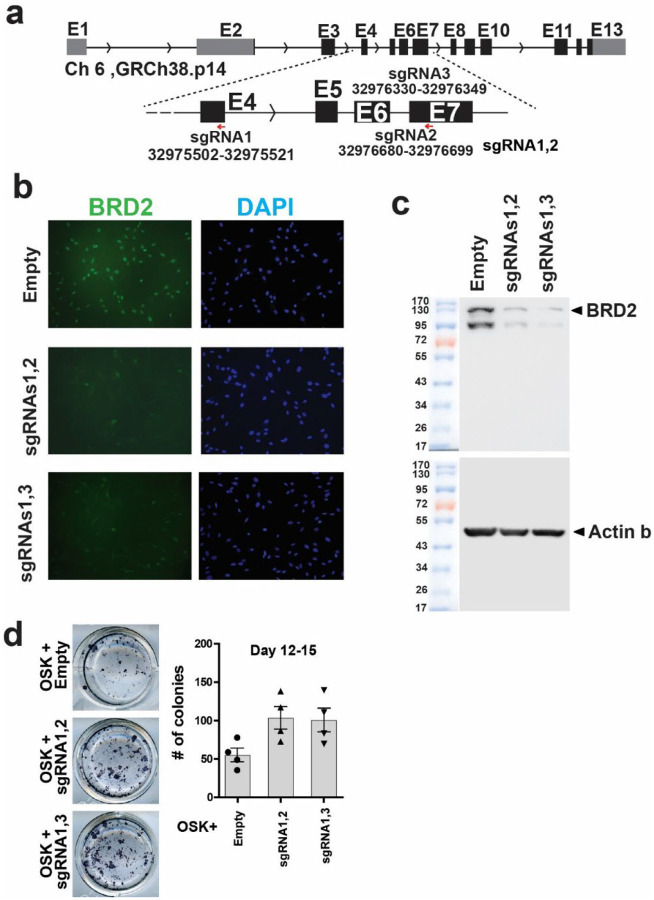
BRD2 disruption by CRISPR-Cas9 benefits iPSC reprogramming in the early stages. **a,** The locations of the target sequences for the corresponding small guide RNA (sgRNA) used in inducible CRISPR knockout of human BRD2. **b,** Immunocytochemistry using a BRD2-specific antibody that shows the reduction of BRD2 signal after activation of Cas9 with doxycycline for two combinations of sgRNAs targeting human BRD2. Inducible Cas9 human fibroblast line was transduced with a pair of sgRNAs on lentiviral vectors. **c,** Validation of BRD2 gene disruption with western blot. Protein extracts were prepared 72 hours after doxycycline-mediated Cas9 induction. **d,** Enhanced iPSC reprogramming at early stages (at day 12 to 15 of reprogramming) upon BRD2 KO by inducible Cas9 vector and constitutive sgRNAs. Left is representative images of ALP positive colonies; Right is the bar plots of colony numbers. n = 3; t-test.

We further designed CRISPR knockout constructs and established a human fibroblast line with doxycycline (dox) inducible Cas9 (Extended Data Fig. 2a). Two combinations of the three sgRNAs resulted in efficient disruption of *BRD2* in the reprogramming starting cells (Extended Data Fig. 2b, 2c). With this system, we observed larger and more colonies before day 15 (day 12 to 15). After this point, however, the HiPSC colonies started to differentiate, and we cannot establish stable HiPSC lines from our *BRD2* KO experiments, indicating distinct roles of BRD2 in pluripotency establishment and maintenance.

**Extended Data Fig. 3 |:**
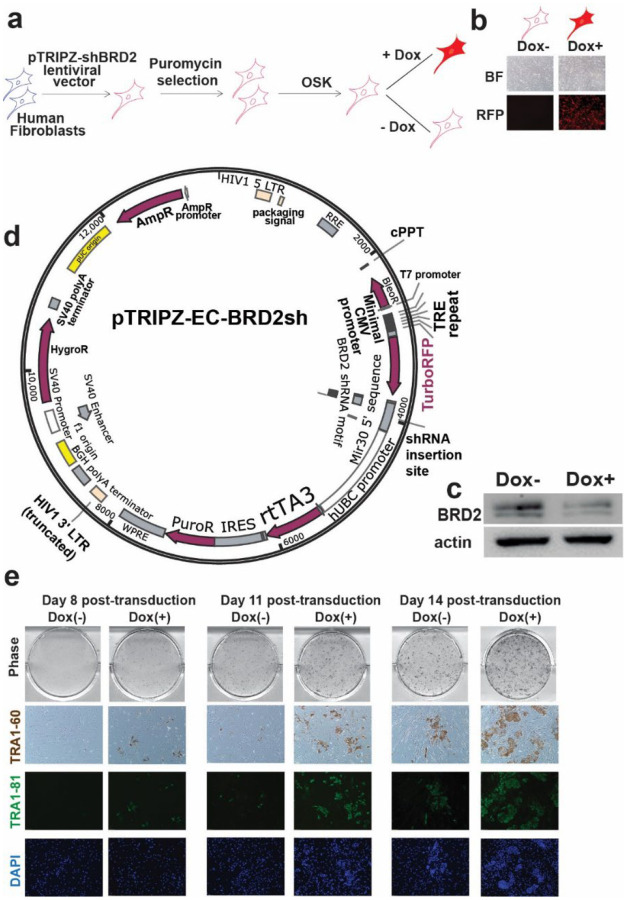
iPSC reprogramming of human fibroblasts with an inducible shRNA against *BRD2*. **a,** Scheme for establishing BJ cell subclones with inducible BRD2 shRNA knockdown by puromycin selection in human fibroblasts as starting cells for reprogramming. **b,** Microphotographs showing the induction of RFP in human BJ fibroblasts transduced with the lentiviral vector pTRIPZ-shBRD2 after selection with puromycin. **c,** Western blot verification BRD2 knockdown through the induction of the shRNA against human *BRD2* by the addition of Doxycycline. Protein was extracted 72 h post induction. **d,** Schematic of TRIPZ all-in-one knockdown construct. RFP is co-expressed along with the mir30-based shRNA. The rtTA protein expression is driven by the human ubiquitin C (hUBC) promoter. **e,** Microphotographs of the reprogramming fibroblasts transduced with OSK and with (Dox+) or without (Dox−) doxycycline addition at days 8, 11 and 14 post-transductions. Representative phase contrast, ICC pictures of TRA-1–60 and IF staining of TRA-1–81 are shown. Scale bar, 100 μm.

We previously showed that pharmacological BET inhibition promotes iPSC reprogramming in the early stages^17^. We then designed inducible *BRD2* shRNA knockdown (Extended Data Fig. 3a, 3d, Extended Data Table 2). Upon dox induction, *BRD2* was efficiently knocked down (Extended Data Fig. 3b, 3c). Induced knockdown of *BRD2* increased numbers of TRA-1–60^+^ and TRA-1–81^+^ cells on day 8 and 11 of reprogramming (Fig. 1h, Extended Data Fig. 3e). Late knockdown (day 11 to 15) had little impact on reprogramming; *BRD2* knockdown in the middle and middle to late stages increased the colony number but did not pass the significance tests (Fig. 1i). Early knockdown (day 3 to 7), however, significantly increased reprogramming (Fig. 1i). Similar to chemical BET inhibition^17^, continuous knockdown from day 3 to 11 or 15 demonstrated the highest reprogramming activities (Fig. 1i). In summary, BRD2 is an early reprogramming barrier, and this barrier lingers at least for the first 10 days.

**Extended Data Fig. 4 |:**
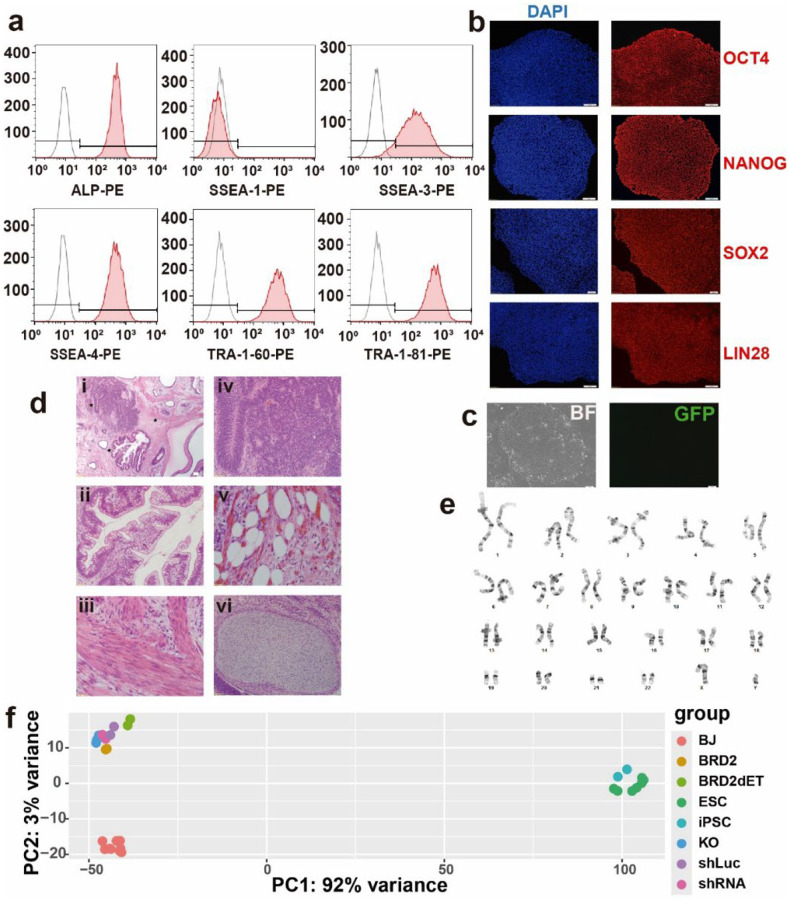
Characterization of iPSCs reprogrammed by shRNA against *BRD2*. **a,** Flow cytometry analyses of the human pluripotent stem cell surface markers TRA-1–60, TRA-1–81, SSEA3, SSEA4, and Alkaline Phosphatase (ALP) along with the negative surface marker SSEA1. **b,** Immunostaining of the iPSCs using antibodies against human OCT4, NANOG, SOX2 and LIN28. Scale bar, 50 μm. **c,** Microphotograph showing the silencing of the reprogramming transgenes as indicated by lack of GFP, which was co-expressed with the reprogramming factors. Scale bar, 100 μm. **d,** H&E staining of paraffin-embedded sections of a teratoma. i) Low magnification (4×; scale bar, 50 μm) of a teratoma section showing three germ layer tissues in one section as detailed in ii (20×), iii (40×) and iv (20×). The three enlarged areas are indicated with stars in i. ii) Gut-like structures (endoderm). iii) Smooth muscle (mesoderm). iv) Neural-like tissue (ectoderm). v) Adipose and connective tissue (40×). vi) Cartilage (20×). **e,** G-banding assay showing normal karyotype. Cytogentic analyses were performed on 20 G-banded metaphase iPSCs. **f,** RNA-seq PCA plot of various cells showing tight clustering of BRD2-shRNA iPSCs with human ESCs. Number of RNA-seq samples, BJ fibroblasts = 9, ESC = 7; all others, n = 2.

We were able to establish HiPSC line with *BRD2* shRNA (shBRD2iPSCs) although we could not do so with *BRD2* KO. The shBRD2iPSCs are pluripotent based on pluripotency marker expression (Extended Data Fig. 4a, 4b), silence of the reprogramming factors (Extended Data Fig. 4c), teratoma formation (Extended Data Fig. 4d), and a pluripotency transcriptome (Extended Data Fig. 4f). The shBRD2iPSCs maintain a normal karyotype (Extended Data Fig. 4e).

### BRD2 is the predominant BET species in the starting cells

**Extended Data Fig. 5 |:**
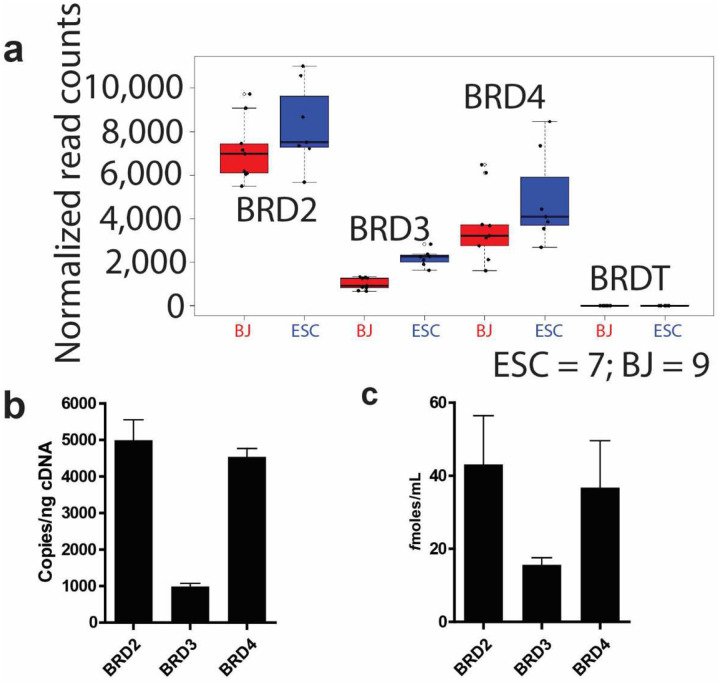
BRD2 is the predominant species in both human fibroblasts and ESCs. **a,** Boxplot of the normalized read counts of BET species in RNA-seq, ESC = 7; BJ = 9 **b,** Quantitative reverse transcription PCR (qRT-PCR) of *BRD2*, *BRD3* and *BRD4*; expression level was determined on BJ cells at 3 different passages using a standard curve for each target gene. **c,** Protein quantitation using competitive sandwich ELISA on BJ cells protein extracts at 3 different passages.

The mammalian BRD2, BRD3 and BRD4 are ubiquitous proteins and the current BET chemical inhibitors are pan-BET. Given that mild BET chemical inhibition enhanced HiPSC reprogramming (Extended Data Fig. 1)^17^, we wondered why *BRD2* shRNA knockdown enhanced HiPSC reprogramming whereas knockdown of *BRD3* and *BRD4* did not. We first reviewed our RNA-seq data. In line with the fact that *BRDt* expression is restricted to gonad, our RNA-seq data showed no expression of *BRDt* in both fibroblasts and PSCs (Extended Data Fig. 5a), but high normalized read counts for the three ubiquitous members. Interestingly, *BRD2* demonstrated the highest expression in human fibroblasts while *BRD3* displayed the lowest expression. *BRD4* expression is half that of *BRD2*. The same trend was observed in human PSCs. Our quantitative RT-PCR confirmed that *BRD2* displayed the highest expression while *BRD3* showed the lowest expression (Extended Data Fig. 5b). Further ELISA quantification using BET protein standards showed similar results to that of RT-qPCR and RNA-seq (Extended Data Fig. 5c).

### *BRD2* shRNA legitimately down-regulates genes of matirsome and its regulation

To reveal the underlying molecular underpinnings of reprogramming enhancement by *BRD2* knockdown, we sequenced RNA (RNA-seq) on the reprogramming cells with and without *BRD2* knockdown. There were 1,970 differentially expressed genes (DEG) upon *BRD2* knockdown indicating a broad and significant transcriptional impact of BRD2 on the reprogramming cells. Of those DEG, the majority (1,163 genes, 59%) was significantly downregulated by *BRD2* shRNA in agreement with its reported positive role in transcription. However, the fraction of up-regulated genes (805 genes, 40.9%) is noteworthy. We previously also observed a large fraction of up-regulated genes for JQ1 inhibition of BET proteins in the reprogramming cells^17^.

**Fig. 2 |:**
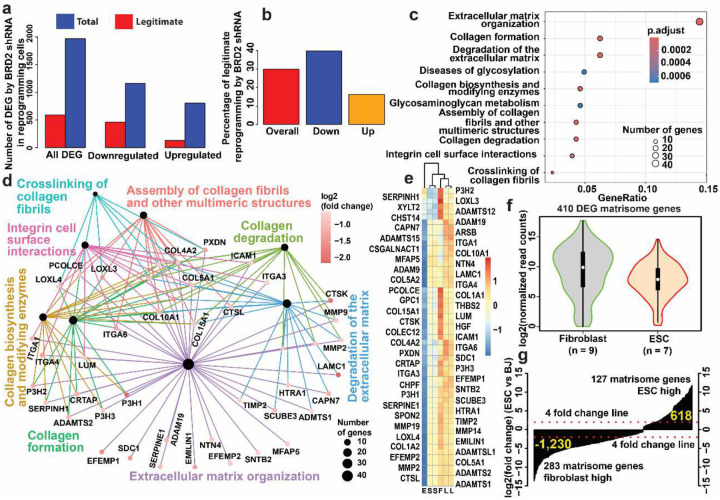
BRD2 knockdown legitimately reprograms genes of matrisome and its regulation as revealed by RNA-seq. **a**, Number of DEG and legitimate reprogrammed genes by *BRD2* shRNA. **b**, percentage of legitimate reprogramming by *BRD2* shRNA. **c**, top 10 enriched pathways for the legitimately down-reprogrammed genes. **d**, cnetplot for the top 8 enriched pathways of the legitimately down-reprogrammed genes. **e**, down-regulation of 57 matrisome and related genes by *BRD2* shRNA towards their pluripotent levels. F, fibroblasts, n = 9 RNA-seq; S, *BRD2* shRNA, n = 2; L, luciferase shRNA, n = 2; E, embryonic stem cells, n = 7. **f**, violin plots for the 410 DEG matrisome genes between human ESC and fibroblasts. **g**, Waterfall plot on log2(fold changes) for the 410 DEG matrisome genes.

We then evaluated the reprogramming legitimacy of those DEG^33,34^. Overall, 30% (592 out of 1,970 genes) DEG underwent legitimate reprogramming (Fig. 2a, 2b). More downregulated genes were legitimately reprogrammed toward the lower pluripotent levels (39.7%, 462 out of 1,163). Only 16.2% (130 out of 805 genes) of the upregulated genes were legitimately reprogrammed upward to the higher pluripotent direction. In sum, *BRD2* knockdown predominantly led to legitimate down-reprogramming of somatic genes, in agreement with our previous chemical BET inhibition in pluripotency reprogramming^17^ and the reported chemical neuron reprogramming of mouse fibroblasts^23^.

**Extended Data Fig. 6 |:**
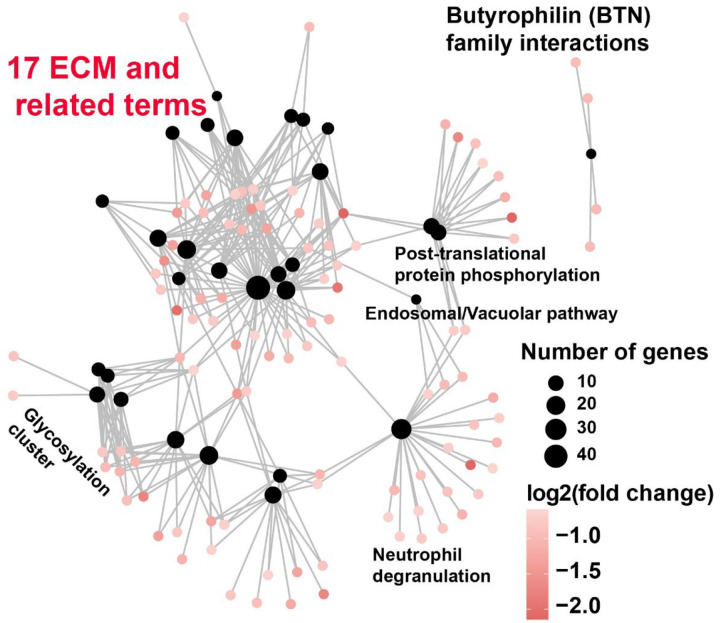
Matrisome and related pathway GO terms form the predominant clusters among all 30 enriched pathway terms for the legitimately down-reprogrammed gene list by *BRD2* shRNA knockdown. Each filled black dot represents a pathway GO term. Each colored graded dot represents one gene with its expression levels as indicated by the color codes. Gene symbols are not labelled for clarity. Selected pathway terms are marked. The 17 terms of the ECM and ECM-related are highlighted as red group label. For a complete list of enriched pathway terms, see Extended Data Table 1.

To understand the nature of the predominantly legitimate down-reprogramming of the somatic program, we ran a pathway enrichment analysis for the 462 legitimately down-reprogrammed genes. The top 10 enriched pathway are overwhelmed by terms of the extracellular matrix (ECM) theme (Fig. 2c) and those ECM terms shared many genes as revealed by cnetplot of the top 8 terms (Fig. 2d). Of note, 17 out of the 30 enriched pathway terms are in the category of matrisome or are related to matrisome (Sheet in in Extended Data Table 1). The only independent term is “Butyrophilin (BTN) family interactions” (4 genes only) (Extended Data Fig. 6). The remaining 13 terms still include shared genes with the matrisome gene network although those terms are not directly related to the matrisome terms (Extended Data Fig. 6). Fifty-seventy matrisome and matrisome-related genes are legitimately downregulated by *BRD2* shRNA (Fig. 2e). In contrast, among the 93 enriched biological process (BP) terms and 13 pathway terms for the upregulated genes by *BRD2* shRNA, we did not see any enriched ECM or ECM-related terms. Given that, we compared the transcription levels of the Naba matrisome gene set^35^. Of the 1,027 curated matrisome genes, 410 are differentially expressed between ESCs and the starting fibroblasts. Among the 410 DEG, 283 (69%) are expressed at significantly lower level in ESCs but 127 (31%) displayed significantly higher expression in ESCs. We then compared the expression levels for those DEG matrisome gene set as a whole. Overall, human ESCs have a much lower expression of matrisome gene set (Fig. 2f). Waterfall plots^34^ also indicated that ESCs have a much weaker matrisome program (Fig. 2g). In summary, *BRD2* knockdown facilitates legitimate reprogramming of 592 genes and the majority is legitimate downregulation of the somatic genes, particularly the genes of matrisome and its regulation, to the pluripotent state.

### BRD2 negatively regulates transcription of lipogenesis genes

**Fig. 3 |:**
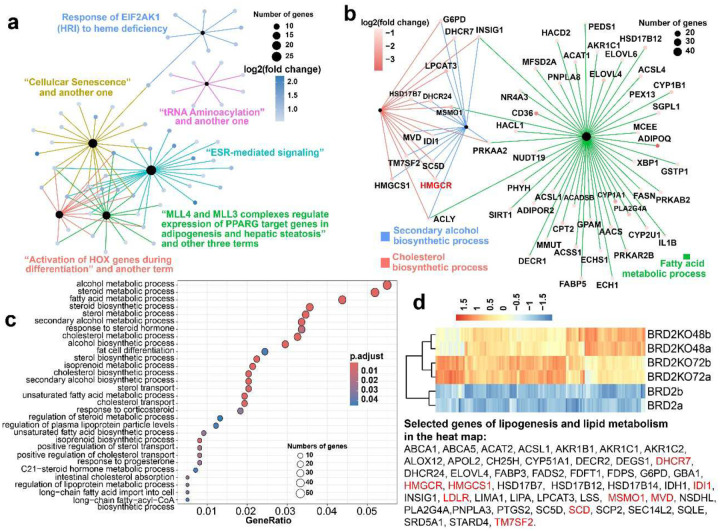
BRD2 negatively regulates lipogenesis and lipid metabolism genes. **a**, cnetplot summarizing all enriched pathways for genes upregulated by *BRD2* shRNA knockdown in the reprogramming cells. **b**, cnetplot for the three lipid-related enriched BP terms for the downregulated genes by BRD2 overexpression in the reprogramming cells relative to *BRD2* shRNA knockdown. Black dots in a and b represent GO categories. Each colored dot represents one gene with its relative expression level. **c**, dotplot for 30 enriched BP terms in the categories of lipid biosynthesis, metabolism and regulation for the genes compromised by BRD2 (n = 2) overexpression in reprogramming cells in reference to *BRD2* KO (n = 4). **d**, Heatmap for the 145 lipid genes suppressed by BRD2 in the reprogramming cells. Selected genes are listed beneath the heatmap, and the full list of genes can be found in Extended Data Table 2. BRD2KO48 and BRD2KO72, BRD2 KO treatment at 48 and 72 hours post induced KO in the reprogramming cells, respectively.

Next, we conducted gene ontology (GO) analyses of the upregulated gene list (805 genes) by *BRD2* shRNA. Because too many enriched BP terms (93) were observed, we focused on reactome analyses of the upregulated genes. In total, much fewer pathway terms (13) are enriched statistically. Intersection and cnetplot analyses indicate that several terms share the exact sets of genes. We, thus, reduced the number of enriched terms to 6 by removing the redundant terms. Cnetplot analyses revealed that the enriched pathways formed three clusters. One term (“tRNA aminoacylation”) was independent of others (magenta in Fig. 3a). The term of “response of EIF2AK1 (HRI) to heme deficiency” share one out of its 7 genes with the “cellular senescence” term. These two independent clusters each include fewer genes (8 each). The remaining four terms are intertwined. “ESR-mediated signaling” and the adipogenesis/steatosis groups shared 11 genes and are semantically related since ESR is of lipid/steroid. This merged steroid/lipid larger group includes 32 genes.

In addition to reprogramming enhancement by *BRD2* shRNA, *BRD2* overexpression almost abolished the iPSC reprogramming (Fig. 1g, and 5a, 5b). We then focused on this dramatic impact of BRD2 on reprogramming. When the RNA-seq data of the reprogramming cells are compared between *BRD2* overexpression and its shRNA knockdown, only two pathway terms were enriched for the downregulated genes by *BRD2* overexpression relative to its knockdown, “transcriptional regulation by TP53” and “Cholesterol biosynthesis” (data not shown). The P53 pathway is a reported barrier for reprogramming, but our data indicates that BRD2 mitigates but its shRNA worsens the P53 barrier, contradicting BRD2 role as a reprogramming barrier. Our attention now was on “cholesterol biosynthesis” pathway given that *BRD2* knockdown enhanced adipogenesis and ESR-mediated signaling as discussed above. Scrutiny of enriched BP terms for BRD2-downregulated genes revealed that three lipid-related BP terms are enriched: “cholesterol biosynthetic process”, “fatty acid metabolic process”, and “secondary alcohol biosynthetic process” (Fig. 3b). These data indicate that BRD2 impairs cholesterol biosynthesis and FA metabolism.

To further confirm BRD2 role in lipid biology, we compared RNA-seq data of the reprogramming cells between the two extreme conditions: *BRD2* inducible KO and its overexpression. There was a total of 2,354 DEG with similar numbers of up- and down-regulated genes (1,175, 1,179, respectively). The top enriched pathway terms for the upregulated genes by BRD2 are predominantly of the category of cell cycle (data not shown) in agreement with the reported BRD2 role in regulation of cell cycle. On the other hand, the top GO and pathway terms for the BRD2-downregulated genes are about lipid biosynthesis, metabolism and regulation (data not shown). We tallied all lipid BP terms for the genes downregulated by BRD2 overexpression vs KO and found 30 such terms (Fig. 3c, and sheet 2 in Extended Data Table 1) with 145 such genes (Fig. 3d). In conclusion, BRD2 suppresses a large set of genes in lipid biosynthesis and metabolism in the reprogramming cells and also abrogates HiPSC reprogramming when overexpressed.

### SCD and HMGCR overcome BRD2 impairment of reprogramming

**Fig. 4 |:**
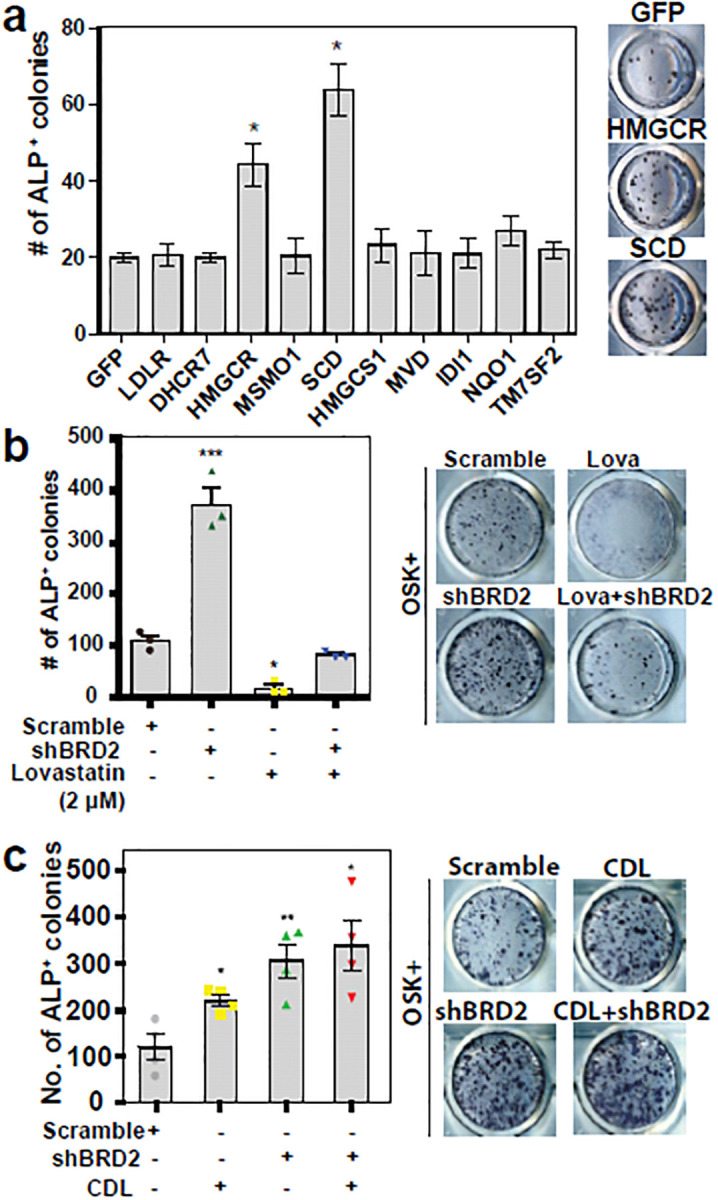
Rate-limiting enzymes of Lipogenesis and lipid supplements overcome BRD2 impairment of reprogramming **a**, Screening of the reprogramming activity of SREBF-regulated metabolic genes. n = 4 independent experiments; *, p < 0.05; t-test. The right side of the bar plots are representative ALP staining images of the reprogramming cells. **b**, The HMGCR inhibitor lovastatin abrogated iPSC reprogramming (day 20, ALP staining). Bar plots are iPSC colony numbers for different conditions as indicated; right panels are representative images of the ALP-stained reprogramming wells. n = 4 independent experiments; *, p < 0.05, t-test. **c**, A chemically defined lipid (CDL) cocktail enhanced iPSC reprogramming (day 20, ALP staining). Bar plots are ALP^+^ colony numbers for conditions indicated; right panels are representative images of the ALP-stained reprogramming wells. n = 4 independent experiments, *, p < 0.05; t-test.

Given that BRD2 suppresses a large set of lipid genes in the reprogramming cells, we cloned 9 such genes (red text in Fig. 3d; Fig. 4a) that are downregulated by BRD2 in the reprogramming cells. We previously reported that HiPSC reprogramming encounters reprogramming stress^22^. We thus include NAD(P)H quinone dehydrogenase 1 (NQO1) because it has antioxidant properties and is upregulated by BRD2 KO by 4.4-fold (data not shown). Our reprogramming experiments showed that SCD and HMGCR enhanced iPSC reprogramming but not NQO1 (Fig. 4a). In contrast, the HMGCR chemical inhibitor (lovastatin) abrogated iPSC reprogramming (Fig. 4b). Given the critical and rate-limiting roles of SCD and HMGCR in lipogenesis, we supplemented the reprogramming medium with lipids. Indeed, lipid supplements promoted iPSC reprogramming (Fig. 4c). Lipid and *BRD2* shRNA demonstrate small synergism (Fig. 4c) indicating overlapping but with some distinct roles. This is supported by the fact that *BRD2* shRNA restored some reprogramming activity in the presence of HMGCR inhibitor (Fig. 4b).

### BRD2 ET domain is the major intramolecular regulator of reprogramming, lipogenesis and matrisome

**Fig. 5 |:**
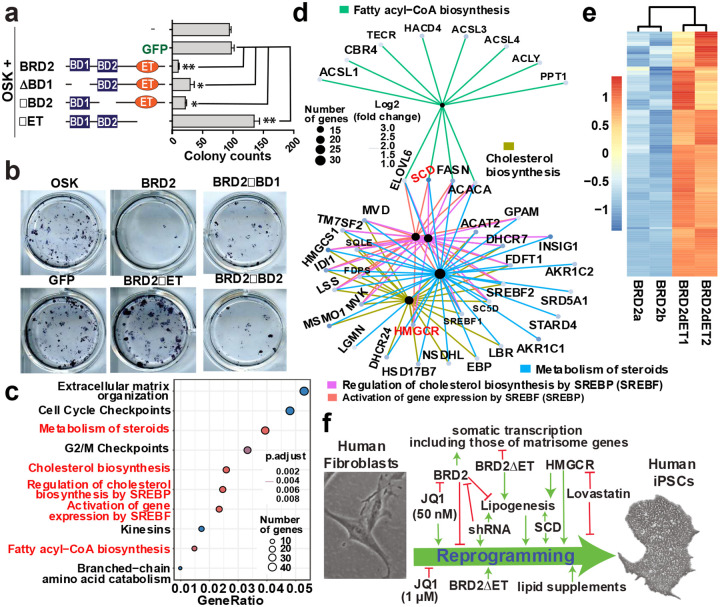
The ET domain of BRD2 mediates the suppression of lipid genes in the reprogramming cells. **a,** Schematic of individual deletion of the three BRD2 characteristic domains and summary of their reprogramming activities. n = 3 independent experiments; ANOVA test was used. **b,** Representative images of ALP staining of the reprogramming wells under different BRD2 domain deletions as labeled. **c**, Dot plot of the top 10 enriched pathway GO terms for the gene list upregulated by BRD2ΔET relative to BRD2 in reprogramming cells. The lipid-related terms are in red text. **d**, cnetplot of the top 5 enriched pathway terms for gene list upregulated by BRD2ΔET relative to BRD2 in the reprogramming cells. **e**, Heat maps of the 119 lipid genes (combined BP and reactome pathway terms) upregulated by BRD2ΔET in reprogramming cells as compared to BRD2 WT. **f**, Schematic summary for BRD2-centered molecule roles in HiPSC reprogramming. Green arrows, enhancing activities; red T or red reverse T, suppressing activities.

Previously, we reported that a truncated isoform of BRD3 (BRD3R) lacking its ET tail promotes the Yamanaka reprogramming^21^. We have also shown that deletion of the three characteristic domains releases reprogramming activity from BET proteins including BRD2^22^. We want to know whether any of these three domains is more critical in terms of reprogramming inhibition. For this purpose, we deleted each individual domain (Fig. 5a) and tested their reprogramming activity. The reprogramming remained significantly lower than the GFP control when each bromodomain was deleted alone, but ET deletion alone significantly increased the reprogramming (Fig. 5a, 5b). In addition, colonies from the *BRD2ΔET* condition were larger as compared to that of the controls and had a more rounded morphology (Fig. 5b).

**Extended Data Fig. 7 |:**
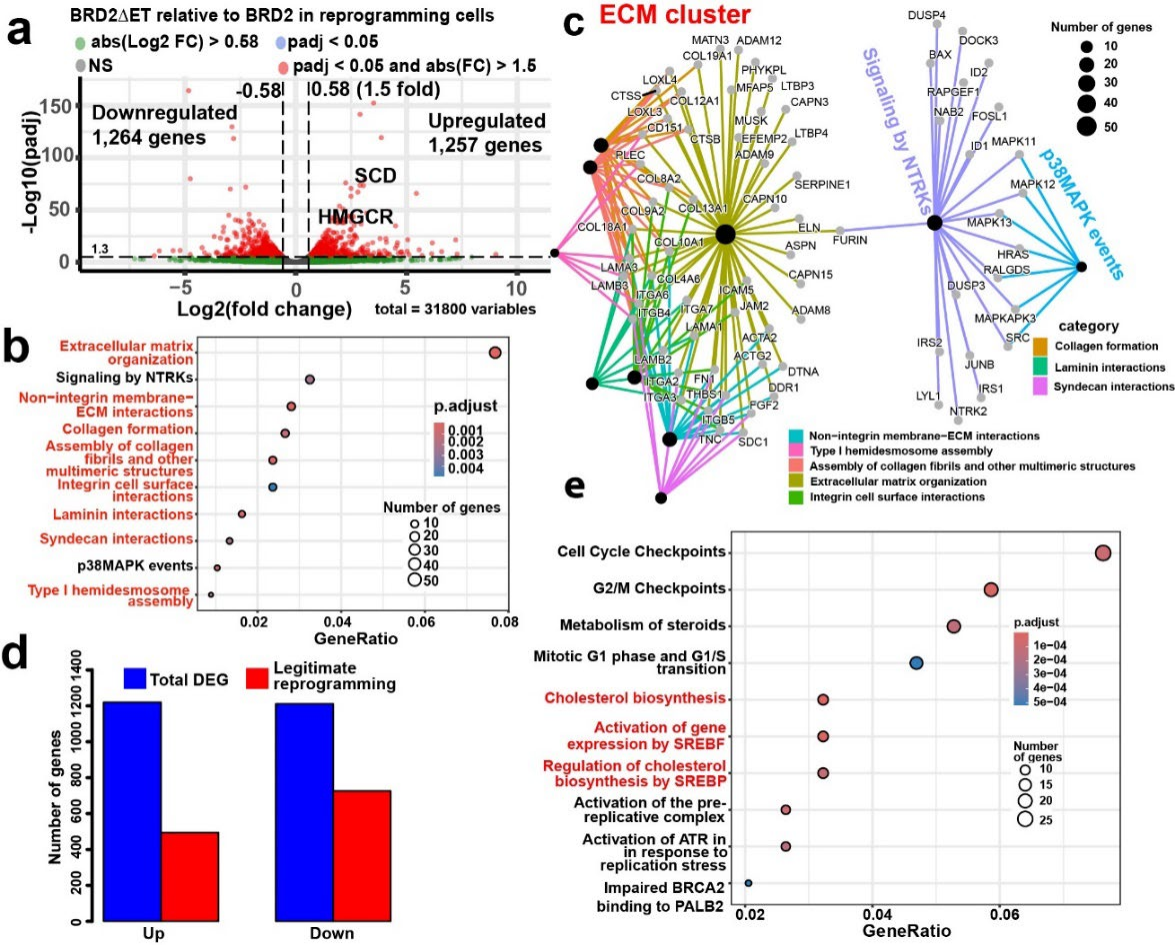
BRD2 ET tails maintain somatic ECM-related transcriptional program. **a**, Volcano plot for RNA-seq on reprogramming cells with overexpression of BRD2ΔET vs BRD2. **b**, dot plot for the top 10 reactome terms of the downregulated genes by BRD2ΔET relative to BRD2 in reprogramming cells. **c**, cnetplot of the top 10 pathway terms in panel B. **d**, Number of genes underwent legitimate reprogramming by BRD2ΔET relative to BRD2. **e**, Dotplot of the top 10 reactome GO terms for to the legitimately up-regulated genes by BRD2ΔET relative to BRD2.

Given ET inhibition of reprogramming observed above, we investigated the transcriptional impact of the ET tail in the reprogramming cells. Compared to BRD2, BRD2ΔET resulted in 2,521 DEG (Extended Data Fig. 7a). Similar amounts of genes are upregulated as downregulated (1,257 vs 1,264), indicating that ET tail plays both positive and negative roles in transcription. Reactome pathway analysis of the upregulated genes by BRD2ΔET relative to BRD2 indicated that five of the top 10 terms are related to lipids (Fig. 5c), and 9 of the top 10 enriched BP terms are related to lipids (data not shown). Cnetplot analysis showed that lipid terms are the most enriched network and all the top 5 pathway terms in cnetplot are related to lipids (Fig. 5d). For the enriched BP terms of the upregulated genes by BRD2ΔET, there were 11 lipid-related BP terms after removing the redundant terms (sheet 3 in Extended Data Table 1). In total, 119 genes in lipid pathways are de-repressed by BRD2ΔET (Fig. 5e). In sum, BRD2 ET tail is an intramolecular inhibitor for transcription of lipid related genes.

We then conducted reactome pathway analysis for the genes downregulated by BRD2ΔET relative to BRD2. Among the top 10 reactome terms, 8 are related to ECM (Extended Data Fig. 7b, 7c). This result is perfectly aligned with observations from *BRD2* shRNA and its KO.

We then examined the reprogramming legitimacy of the DEG by BRD2ΔET. Out of the 1,120 up DEG, 494 (40.5%) are expressed significantly higher in ESC, representing a legitimate up-reprogramming; on the other hand, 726 out of the 1,221 down DEG (59.5%) underwent legitimate down-reprogramming (Extended Data Fig. 7d). We then run GO analyses for the legitimate reprogramming gene lists. Among the 15 enriched reactome terms for the legitimate down-reprogramming gene list, 13 are in the category of ECM. The remaining three terms are associated with 9 independent genes only (data not shown). For the legitimate up-reprogramming genes, the top reactome terms are related to cell cycle (Extended Data Fig. 7e) as we reported before^21,22^. Three enriched reactome terms of lipid biosynthesis are among the top 10 legitimate reprogramming terms (Extended Data Fig. 7e). In sum, BRD2 ET tail impairs iPSC reprogramming and suppresses the gene transcription of lipid biosynthesis in the reprogramming cells. BRD2 ET tail, on the other hand, positively regulates transcription of somatic genes, in particular, ECM-related genes.

### BRD2 binds to genes of lipogenesis and regulates their epigenetics

**Extended Data Fig. 8 |:**
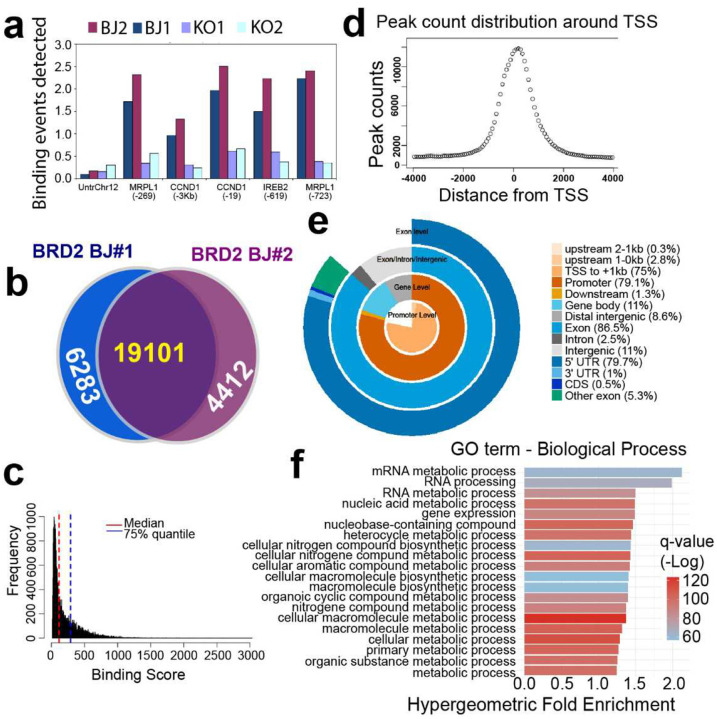
Overview of BRD2 ChIP-seq **a**, ChIP-PCR demonstration of known BRD2 binding sites in extracts of 30 μg chromatin from two BJ control fibroblasts samples and two BRD2 KO fibroblasts (transduced with sgRNA Cas9 vectors), using the anti-BRD2 antibody A302–582A (Bethyl-Fortis). Primers of desert sequence in an untranscribed region in chromosome 12 were used as a negative control. **b,** Venn diagram showing replicate concordance. Only overlapping peaks (yellow) were used for subsequent analyses. **c**, Binding score distribution of the 1,9101 common BRD2 peaks. Marked are the median and 75% quantile of the scores. Peaks above the 75% quantile score were regarded as strong bindings. **d**, Peak count distribution of BRD2 strong binding sites around TSS. **e**, Genomic element distribution of BRD2 strong interactions. 75% of the peaks are located within 1 kb around TSS. **f**, List of top 20 enriched GO terms (Biological process) found using GREAT (https://great.stanford.edu/great/public/html/).

**Fig. 6 |:**
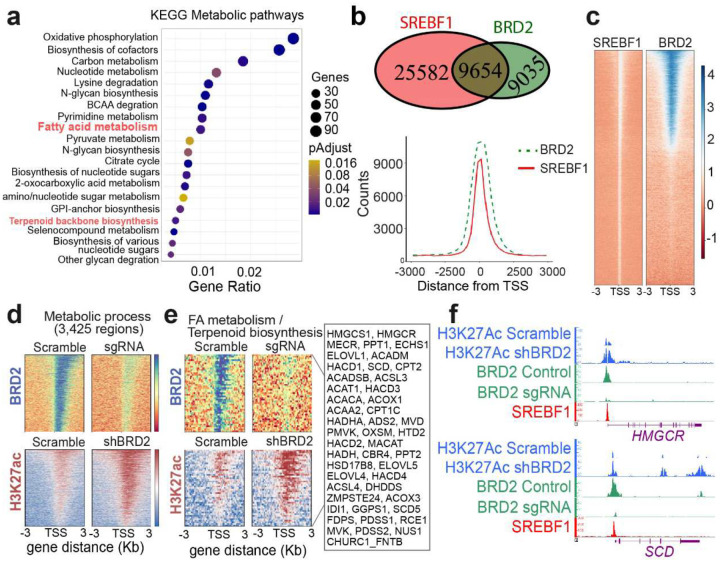
BRD2 directly binds to promoters of metabolism genes including lipid metabolism and negatively regulates their H3K27Ac status. **a**, Top 20 KEGG terms of the category of “metabolism” enriched in the set of genes with strong BRD2 binding. **b**, BRD2 and SREBF1 share 9,654 binding regions. Upper, Venn diagram of SREBF1 and BRD2 binding sites (all binding regions are included). Lower, counts of binding sites around TSS. **c**, Heatmaps of SREBF1 and BRD2 binding regions showing all SREBF1-specific regions close to TSS. **d**, ChIP-seq read density heatmaps of BRD2 and H3K27Ac marks at metabolic related genes associated with the GO terms of metabolic processes found in annotated BRD2 interaction regions close to TSS. **e**, ChIP-seq normalized read density heatmaps of BRD2 and H3K27Ac marks at TSS of genes associated with the KEGG enriched pathways “Fatty acid metabolism” and “Terpenoid biosynthesis” shown in A, that were annotated with strong BRD2 bindings. **f**, ChIP-seq normalized read density tracks of BRD2 (green), H3K27Ac (blue) and SREBF1 (red) on the *HMGCR* and *SCD* genes. A side-by-side comparison between *BRD2* shRNA (shBRD2), or sgRNA and scrambled control was included.

To gain some epigenetic insights into our findings above, we conducted ChIP-seq with a BRD2 antibody. As expected, ChIP PCR with our antibody can pull down the target sequences of the known BRD2-regulated genes (Extended data Fig. 8a). Intersection analysis indicates that our ChIP-seq duplicates share majority of the binding peaks (19,101, 64%). To increase credibility, we used the 19,101 common peaks only in the analyses below. We considered those peaks with binding scores above 75% quantile (Extended Data Fig. 8c and 8d) to be strong BRD2 binding. Seventy-five percent of the strong binding peaks are within 1kb surrounding TSS (Extended Data Fig. 8e). Of the BRD2 strong binding sites, KEGG terms of “fatty acid metabolism” and “terpenoid backbone biosynthesis” are enriched (Fig. 6a). These data agree with our RNA-seq observation that BRD2 regulates genes of lipid metabolism. SREBF1 is the master transcription factor to regulate genes of cholesterol and FA biosynthesis. Indeed, BRD2 and SREBF1 co-bind 9,654 sites (Fig. 6b and 6c). We then focused on the gene set of “metabolic process” and found that BRD2 binds to those genes and the binding become significantly weaker upon *BRD2* disruption indicating specific of our antibody (Fig. 6d, upper panels). Unexpectedly but interestingly, upon *BRD2* knockdown, the H3K27Ac marks were increased substantially on those genes (lower panel, Fig. 6d). We further analyzed binding on genes of “fatty acid metabolism” and “terpenoid biosynthesis”. Similarly, BRD2 binding decreased significantly upon its disruption, and the active transcription mark H3K27Ac increased significantly upon BRD2 disruption. This is in line with our RNA-seq data that showed a negative regulation of lipogenesis genes by BRD2. We then examined the promoters of *HMGCR* and *SCD*. BRD2 and SREBF1 co-bind both promoters. Upon *BRD2* disruption, H3K27Ac mark was increased on both *SCD* and *HMGCR* (Fig. 6f). These ChIP-seq data support our previous conclusion of BRD2 suppression of lipogenesis revealed by our RNA-seq data.

## Discussion

We have previously identified that the short isoform of human BRD3 lacking its ET-containing tail enhances HiPSC reprogramming^21^. We later found that the pan BET chemical inhibitors promote HiPSC reprogramming at low concentrations while the widely used high concentrations impair it^17^. The present study shows that BRD2 appears to be the target of the beneficial mild chemical inhibition in promotion of reprogramming (Fig. 5f). Pan BET chemical inhibition enhances mouse fibroblast reprogramming to functional neurons^23^. Liu *et al*. reported that BRD4 enhanced mouse iPSC reprogramming^24^, but it might do so only at the late stage and another group showed that early BRD4 inhibition slightly increases mouse fibroblast reprogramming to iPSCs^25^. The latter report, however, did not examine shRNA against *BRD2* and *BRD3*^25^. Using shRNA against each of the expressed BET members, we found that only BRD2 acts as a reprogramming barrier. It is important to recognize that all other BET studies have used mouse cells^23–25^ while our investigation focused on human iPSC reprogramming. Species might be sources of some discrepancies.

We reported here that *BRD2* shRNA and dominant negative (DN) mutants legitimately downregulated somatic genes of matrisome and matrisome regulation towards the lower pluripotent levels. Human PSCs appear to have a unique matrisome^15^. Human ESC culture needs the support of feeder cells^13^. In feeder-free systems, human PSCs still require coating of growth surface with matrix proteins^14^. Human PSC passage is also special in that the traditional trypsin detachment kills hPSCs^36^, indicating a unique cell-matrix or cell-surface environment. In addition, hPSCs rely on cell-cell contacts for self-renewal and form tight colonies in culture^36^. Individualized hPSCs die readily. Of notes, the Yamanaka factors downregulate somatic transcriptional program. Given this, BRD2 role in maintaining the somatic transcriptional program including genes of matrisome may be masked to some extent by the Yamanaka factors. Nevertheless, with our concept of reprogramming legitimacy we can still demonstrate that BRD2 maintains the somatic program and is thus an adverse factor of pluripotency reprogramming.

We showed here that lipogenesis deficiency is a reprogramming barrier. This aligns with the fact that PSCs have a truncated and fast cell cycles as compared to the somatic ones, and establishment of this short pluripotent cell cycle is part of iPSC reprogramming^37^. The fast-proliferating iPSCs require more lipids as the key cellular building blocks of new cellular membrane. In support of our results, lipids regulate genome integrity, self-renewal and pluripotency in PSCs^38^. Our data showed that the SCD desaturase enhanced HiPSC generation. This is in line with the report that mouse ESCs have significantly higher monounsaturated FA (18:1, 20:1) than fibroblasts^39^, and that 4–5 unsaturated cardiopilin, a major component of mitochondria inner membrane, is more abundant in mouse ESCs^39^. In both human and mouse blastocysts, unsaturated lipids and membrane fluidity increased as compared to that at the 2-cell to 8-cell stage embryos^40^. SCD regulates the membrane fluidity and cell polarity of the embryonic cells in the pre-implantation embryos^40^. A recent report suggests that *de novo* lipogenesis is active between mouse 8-cell and pre-implantation embryos when lipid supply is not available from oocyte reserves or the pregnant mother^28^. Those authors also showed that *de novo* lipogenesis is active in human PSCs cultured in the chemically defined E8 medium.

Interestingly, our data suggests that BRD2 suppresses lipogenesis even though it is generally considered as a positive transcriptional regulator. Our BRD2 and H3K27Ac ChIP-seq data support our RNA-seq observation that BRD2 suppresses gene expression of lipogenesis and lipid metabolism but our observed increase of H3K27Ac marks on those genes upon BRD2 loss challenges the current paradigm about relationship of BET proteins and acetylation. In support of paradigm-changing findings here, a previous study indicates that mice with lower BRD2 develop more fat indicating BRD2 suppression of lipogenesis in animals^41^.

Here, we propose that BRD2 impedes HiPSC reprogramming largely by suppressing lipogenesis (Fig. 5f). This does not exclude other mechanisms (Fig. 5f). We do see that *BRD2* shRNA and BRD2ΔET legitimately down-reprogram the somatic genes, especially those of matrisome and its regulation. However, those positive impacts may not be seen phenotypically by the commonly used measurements of reprogramming efficiency^42^. We previously proposed that BET mini proteins enhanced reprogramming by mitigating reprogramming stress^22^. We do see that many stress response GO terms are among the enriched lists for DEG by *BRD2* shRNA and BRD2ΔET. However, the mechanism of lipogenesis deficiency to explain BRD2 as a reprogramming barrier is clearly supported by genetic, pharmaceutical, and lipid supplement data with the rate-limiting lipogenesis enzymes of SCD and HMGCR. One future direction is to investigate whether lipogenesis deficiency has any relationship with reprogramming stress. It is well known that lipids are targets of various cellular stress. For example, lipids are one of the major targets of oxidative stress^43^. ER stress alters lipid gene expression since ER is the major site of lipogenesis. ER stress pathway and lipogenesis also crosstalk^44^.

## Online Methods

### Production of lentivirus particles

Lentivirus vectors were generated using PEI-mediated transfection into Lenti-X^™^ 293T (Takara, Cat. 632180) cells. Briefly, 1×10^7^ Lenti-X^™^ 293T cells were seeded into one 150mm dish per vector and cultured in 24–30 mL expansion medium: DMEM-F12 (Gibco, Cat. 12400–024) supplemented with 10% FBS (Gibco, Cat. 10437–028) to 80%-90% confluence. At 80%-90% confluence, medium was replaced with 24 mL fresh expansion medium at least 2 hours before transfection. Transfection solution was prepared by mixing a 3 mL plasmid solution, containing envelope, packaging and transfer plasmids at a ratio of 1:3:4 (total amount of 60 μg of plasmid), with a 3 mL “PEI solution”, containing 60 μg/mL of polyethylenimine (PEI, Polysciences Inc., Cat. 24765–2). The DNA:PEI ratio is 1:3 by weight. Plasmid and PEI solutions were prepared in DMEM/F12 and mixed to get a final transfection solution of 6 mL. The transfection solution was incubated for 15 minutes at room temperature and added dropwise into the cell cultures of 80–90% confluence. The cultures were incubated for 16 hours at 37 °C, 5% CO_2_. After the transfection, the medium was replaced with 20 mL fresh complete expansion medium, and the cells were incubated for 72 hours. Medium with lentiviral particles was filtered using Stericup low-protein-retention 0.45-μm PVDF membrane filters (Millipore). The viruses in the filtered media was concentrated first by the addition of PEG6000 (8.5% final concentration) and NaCl (0.4M final concentration), followed by an incubation at 4 °C for 3 hours with periodical mixing. The virus-PEG complex was then centrifuged at 4,500×g for 45 minutes. The resulting lentiviral pellet were resuspended into 150 μL sterile PBS. This virus concentrate was aliquoted and stored in small volume at −80 °C. Titration of lentiviral concentrates was performed by transducing HeLa cells and analyzing the co-expressed GFP signal by flow cytometry (Fortessa Flow Cytometer, BD) 72 hours later as described^36^.

#### Human fibroblast reprogramming into hiPSCs^36^

BJ fibroblasts (ATCC, CRL-2522) at passages 3 to 6, were seeded into 12-well plates at a density of 6.5×10^4^ cells/cm^2^. Twenty-four hours after plating, OCT4, SOX2 and KLF4 (OSK) lentiviral vectors, or OCT4, SOX2, KLF4 and MYC (OSKM) lentiviral mix were added to the fibroblast culture in expansion medium supplemented with 5 μg/ml hexadimethrine bromide. When indicated, a shRNA lentiviral vector targeting *BRD2*, *BRD3*, *BRD4* or Luciferase (Scramble control), or a lentiviral vector for the overexpression of BET proteins (BRD2, BRD3, BRD4, or BRD2 deletions) or GFP (mock control), was added into the medium. The cells were transduced for 12 to 16 hours at 37 °C and 5% CO_2_. The next morning, viral particles were removed, and a fresh fibroblast medium was added. Forty-eight hours after transduction, the cells were passaged into Geltrex-coated 12-well or 24-well plates at a density of 5×10^4^ cells/cm^2^. At day 3 post transduction, the cells were cultured with the serum free medium E7 (E8 without TGF beta). From day 3 on, the medium was changed daily. From day 15 on, the cells were cultured with the xeno-free medium E8. At day 20, the colony number was determined by ALP or TRA-1–60 staining. For iPSC isolation, compact colonies were picked up manually and seeded into a Geltrex-coated well of a 24-well plate. HiPSC colonies were subcloned to purify GFP negative cells and establish hiPSC lines with transgenes silenced. The established HiPSC lines were characterized by the expression of pluripotency markers, chromosome stability, transcriptome clustering, and differentiation capacity through teratoma formation assay as described^42^.

#### BET protein quantitation

Total BET protein quantitation was performed using validated sandwich and competitive ELISA kits from MyBiosource (Cat. MBS945281, MBS7245725, MBS7206026). BJ cell protein extracts from 3 different passages were obtained by cell disruption using 1× RIPA buffer plus 1× Halt protease inhibitor cocktail (Thermo Scientific, 78429) followed by sonication. Protein concentration was determined using BCA assay (ThermoFisher Scientific) and diluted to 1 μg/mL. Samples were diluted 1:10 to 1:20 using 1× sample dilution buffer provided by manufacturer. Assay procedure was performed per manufacturer’s guidelines. Standard curve was determined using linear regression from logarithmic transformations of concentration versus OD.

### Alkaline phosphatase (ALP) staining

To quantitate the reprogramming efficiency, colonies that emerged at day 20 after transduction (or indicated time) were stained using BCIP/NBT colorimetric staining technique as reported before^42^. Briefly, ALP staining solution was prepared by diluting bromochloroindolyl phosphate (BCIP) and nitro blue tetrazolium (NBT) in Tris buffered saline, pH 9 – 9.5, at a final concentration of 150 μg/mL and 300 μg/mL, respectively. Cells were fixed with cold methanol for 10 minutes followed by a brief wash with PBS. Then, 500 μl of ALP staining solution was added to each well of a 24- or 12-well plate, followed by an incubation of 10–20 minutes. ALP staining solution was removed and 500 μl of PBS was added to stop the reaction. Colonies were counted using a phase contrast microscope.

#### TRA-1–60 staining

Colonies positive for TRA-1–60 were identified through the highly sensitive chromogenic 3,3' diaminobenzidine assay (Metal-Enhanced DAB Substrate Kit, Thermo Scientific, Cat. 34065) as we reported before^42^. Briefly, cells at day 20 of reprogramming (or indicated time) after transduction were fixed with ice cold methanol for 10 minutes and then blocked with 2% BSA in PBS for 30 minutes. Biotinylated anti TRA-1–60 antibody in blocking solution (eBioscience, Cat. 13886382) was added into the fixed cells and incubated overnight at 4 °C. After antibody removal by 3 washing with PBS, streptavidin-HRP (BD, Cat. 554066) was added and incubated for 30 minutes at 20–25 °C. After washing the cells with PBS, 250 μl of 1× metal enhanced DAB substrate was added to each well and incubated for 10 minutes. After incubation, substrate solution was removed and 500 μL PBS was added. TRA-1–60 positive colonies were counted using a phase-contrast microscope.

#### Immunocytochemistry

The immunocytochemistry has been described in detail previously^42^. Briefly, 8-well chamber slides (Sigma, Cat. C7182) were coated with 1% hESC-qualified Geltrex (Gibco, Cat. A1413302) for 1 hour at 37 °C. Cell were seeded at 50% confluence and cultured with E8 medium until around 80% confluence. Cells were washed with PBS and fixed with 4% paraformaldehyde for 10 minutes. Cells were blocked and permeabilized for 30 minutes, using PBS (Gibco) supplemented with 2% BSA Fraction V (Roche) and 0.3% Triton. Primary antibodies (OCT4, SOX2, NANOG and LIN28) were diluted in the blocking-permeabilization solution. Cells were incubated with the diluted antibody overnight at 4 °C. After washing, cells were incubated with the secondary antibody for 1 hour at 20–25 °C. The cells were washed, and the chamber removed carefully. The cells on slides were mounted using ProLong^™^ Gold Antifade Mountant with DAPI (Invitrogen, P36935).

### Flow cytometry

We harvested iPS cells with TrypLE and resuspended the cells in 1 mL of fluorescence activated cell sorting (FACS) buffer (2% BSA, 2 mM EDTA in PBS). The cell clumps were removed by passing through a cell strainer (70 μm). The individualized cells were collected by centrifugation. Cell pellets were resuspended in FACS buffer. About 2.0 × 10^5^ cells in 100 μL FACS buffer were incubated with a PE-conjugated antibody at 4 °C for 30 min. The cells were then washed three times with 3 mL FACS buffer each wash. Cell pellet was resuspended in 600 μL FACS buffer. Then, 10 μL 7-AAD (20 μg/mL stock) was added before assay with flow cytometer (LSRFortessa; BD Bioscience) equipped with BD FACSDiva^™^ acquisition software. Antibodies used are PE-SSEA3 (#560237, BD Pharmingen), PE-SSEA4 (#560128; BD Pharmingen), PE-TRA-1–60 (#560193; BD Pharmingen), PE-TRA-1–81 (#560161, BD Pharmingen), PE-hAlkaline phosphatase (FAB1448P, R&D), PE-SSEA1 (#560142; BD Pharmingen), PE-mouse IgG1 (#555749, BD Pharmingen), and PE-mouse IgM (#555584, BD Pharmingen).

### Western blot

Fibroblasts were cultured in 6-well plates at a density of 6.5×10^4^ cells/cm^2^. After 24 hours, the cells were transduced with lentiviral vectors for the knockdown or the overexpression of *BRD2*, *BRD3* and *BRD4* as well as with a control vector which targets Luciferase or overexpresses GFP, respectively. Forty-eight hours after transduction, protein was extracted using RIPA buffer supplemented with 1× Halt^™^ Protease Inhibitor Cocktail (Thermo Scientific, 78429). The protein concentration was assessed using BCA Protein Assay Kit (Pierce, Cat. 23225). Twenty micrograms of protein per sample were run using traditional Tris-glycine protein polyacrylamide gel system. After electrophoresis, the protein was transferred to PVDF membranes (Millipore, Cat. IPVH00010) using a Mini Trans-blot Cell (Biorad), set at 90 mA constant current for 16 hours at 4 °C. Target protein was visualized using Supersignal West Pico Plus (Thermo Scientific, Cat. 34580).

#### Quantitative reverse transcription PCR (qRT-PCR)

RNA was first harvested from the monolayer cultures using TRIzol^™^ Reagent (Invitrogen, Cat. 15596026). After precipitation with 70% ethanol, RNA pellet was resuspended in 80 uL of 1× DNAse digestion buffer supplemented with 5 units of DNAseI (Thermo Fisher Scientific, Cat. AM2222). The reaction was incubated for 10 minutes at RT. The reaction was stopped and cleared using *Quick*-RNA Miniprep columns (Zymo Research, Cat. R1054). The RNA was eluted with 50 μL Nuclease-free water and stored at −80 °C. RNA concentration was determined using Nanodrop spectrophotometer (Thermo Fisher Scientific). RNA quality was assessed by agarose-gel electrophoresis and high-resolution electrophoresis. The cDNA was prepared from 1 μg of total RNA, using QuantiTec Rev Transcription kit (Qiagen, Cat. 205311). PCR reaction was prepared using 1× Kapa SYBR Fast kit master mix (Sigma-Aldrich, KK4602), 200 nM forward and reverse primers, and 1 ng/μL cDNA. PCR reactions were run on a ViiA 7 QuantStudio (Applied Biosystems) device and analyzed using QuantStudio Software v1.3 (Applied Biosystems). Primers used are: hBRD2f1, AAT GGC ACA AAC GCT GGA AAA; hBRD2r1, CAC TGG TAA CAC TGC CCTG; hBRD3f1, TGC AAG CGA ATG TAT GCA GGA; hBRD3r1, CAT CTG GGC CAC TTT TTG TAG AA; hBRD4f1, GAG CTA CCC ACA GAA GAA ACC; hBRD4r1, GAG TCG ATG CTT GAG TTG TGTT; hBRDTf1, GAT CAC GAA GTT GTG ACA ATG GC; hBRDTr1, CAA CAG GTT CAA TCG GGA TCTT.

### RNA-seq and analysis

Total RNA was isolated from cell cultures using Trizol followed by RNA clean-up using *Quick*-RNA Miniprep columns (Zymo Research) as described before^22^. RNA was eluted in nuclease free water and sent for sequencing at Novogene. Library was prepared using the NEBNext Ultra II RNA Library Prep by Illumina. Human mRNAs were purified by oligo(dT) beads. Non-stranded protocol was used. The purified mRNAs were fragmented, and cDNA was synthesized using random hexamer primers and the M-MuLV Reverse Transcriptase (RNase H-). The second strand was subsequently generated by dNTPs, DNA polymerase I and RNase H. Double-stranded cDNA molecules were purified by AMPure XP beads and overhanging ends were repaired to blunt ends by exonuclease/polymerase. After 5′ phosphorylation and 3′ adenylation, the cDNAs were ligated with P5/P7 sequencing adapters to prepare for hybridization. To select the insert fragment of 150–200 bp in length, the modified libraries are purified with AMPure XP system (Beckman Coulter, Beverly, USA). Sequencing was conducted on Novaseq6000 S4 Illumina by Novogene using a paired-end protocol (150 bp).

RNA-seq FASTQ files were mapped to human genome hg38 on LoneStar6 at Texas Advanced Computing Center (TACC) using STAR package and paired-end unstranded setting. Reads were counted using featureCounts package on LoneStar6. Read counts were normalized and quantified using DESeq2 R package with *postcount* algorithm for size factor estimation. GO and pathway term analyses were performed using clusterProfiler and ReactomePA R packages. The functional enrichment results are then visualized using enrichplot or GOplot R packages. RNA-seq expression levels are visualized using Pheatmap, Vioplot packages, and the *boxplot()* function of the base Graphics R packages as detailed before^45,46^. Volcano plot was prepared using the R package of EnhancedVolcano.

### *Plasmids and shRNA* knockdown

Plasmids for the overexpression of *BRD2*, *BRD3* and *BRD4* were generated by cloning blunted, PCR amplified ORFs into the vector pLVH-EF1a-AcGFP-2SmaI. Inducible BRD2 shRNA was designed using DSIR siRNA design tool (http://biodev.cea.fr/DSIR/DSIR.html) and cloned into an inducible TRIPZ vector (Horizon) shRNA system^47,48^. Transient shRNA knockdown constructs were purchased from Sigma (Mission^®^ shRNA) or cloned in-house and verified in the authors’ lab (Extended Data Table 2). shRNA was delivered to reprogramming cells via lentiviral transduction.

#### CRISPR-mediated gene disruption

BJ cells were transduced with the pCW-Cas9 doxycycline-inducible lentiviral vector, and positive cells were purified by the addition of 0.5 mg/mL puromycin for 5 days. For disrupting BRD2 gene expression, combinations of validated sgRNAs were used. BRDN0001487068 (Addgene, Cat. #77983), BRDN0001148966 (Addgene, Cat. 77985) (Extended Data Table 2). Each sgRNA was packaged into lentiviral particles, concentrated, and transduced overnight into Cas9^+^ BJ cells. Twenty-four hours after transduction, medium was replaced and supplemented with 1 μg/mL doxycycline. Doxycycline-mediated Cas9 induction was maintained for 24 hours.

### ChIP-seq and analysis

BRD2 ChIP-seq experiment was performed by Active Motif (FactorPath service) using the anti-BRD2 antibody A302–582A (Bethyl-Fortis). For the histone mark H3K27Ac ChIP-seq, we used antibody of MA5–23516 (Invitrogen) and SimpleChip Kit (Cell Signaling Technologies, Cat. #9002) following manufacturer guidelines. Briefly, BJ cells were seeded into six 150 mm dishes and cultured in expansion medium to obtain around 2×10^7^ cells per experiment. Cells were treated with formaldehyde at a final concentration of 1% for 9 minutes. Crosslinking was quenched with glycine at a final concentration of 125 mM. Cells were scrapped and pelleted by centrifugation at 750×g and washed with cold PBS. Cell pellets were processed using SimpleChip Kit (Cell Signaling Technologies, Cat. #9002) following manufacturer guidelines. Chromatin extracted from 5 million cells were used for each ChIP. For reverse-crosslinking, immunoprecipitated samples were incubated at 65 °C overnight in the presence of NaCl and Proteinase K. DNA was isolated using Chip DNA Clean & Concentration (Zymo Research, Cat. D5201). Samples were eluted in 20 μL nuclease-free water and stored at −20 °C until use. Fragment size was assessed using high-performance electrophoresis. All samples were sequenced using Illumina platform and single-end protocol.

FASTQ files were mapped to the human genome GRCh38 with Bowtie2 using single end setting with local alignment. BAM files were filtered by read mapping quality (MAPQ>20). Peak calling was performed using MACS2 package. Overlapping peaks and peak annotations were performed using ChIPpeakAnno package^49^. Peak density heatmaps were generated using Deeptools^50^. Peak coverage was visualized using integrated genome browser (IGB)^51^.

## Extended Data

**Extended Data Table 1 |:** Enriched GO terms upon BRD2 manipulation in the reprogramming cells. **Sheet 1**. List of all enriched pathways for the legitimately downregulated genes by *BRD2* shRNA in the early reprogramming cells. **Sheet 2**. Complete list of 30 GO terms of lipid BP categories for genes downregulated by BRD2 relative to BRD2 KO in the reprogramming cells. **Sheet 3**. Enriched GO terms in the category of biological processes of lipids for the genes with increased expression upon BRD2ΔET expression relative to BRD2 overexpression in the reprogramming cells.

**Extended Data Table 2 |:** Sequence information for shRNA and CRISPR KO.

## Data Availability

RNA-seq and ChIP-seq data were deposited at the Gene Expression Omnibus under accession number of GSE304893 without any restriction for access upon publication of this manuscript. There is no clinical patient data in this study. SREBF1 ChIP-seq data were retrieved from GSE29611 and GSM935627 (SREBF1_1: SRR502659; SREBF1_2: SRR502658; SREBF1_3: SRR502660; Input: SRR353506).
